# Dental Implants in Patients with Osteogenesis Imperfecta – Clinical and Radiographic Outcome in Six Patients

**DOI:** 10.3290/j.ohpd.b3858615

**Published:** 2023-02-02

**Authors:** Frederikke Maria Fogh, Simon Storgård Jensen, Thomas Kofod, Eva Lauridsen

**Affiliations:** a Dentist, Resource Center for Rare Oral Diseases, Copenhagen University Hospital, Denmark. Collected and analysed data, wrote, reviewed and edited the manuscript.; b Consultant, Department of Oral and Maxillofacial Surgery, Copenhagen University Hospital Denmark; Professor, Department of Oral Surgery, Institute of Odontology, Faculty of Health Sciences, University of Copenhagen, Denmark. Concept and methodology, data analysis, reviewed and edited the manuscript, project administration.; c Head and Consultant, Department of Oral and Maxillofacial Surgery, Copenhagen University Hospital, Denmark. Concept and methodology, reviewed and edited the manuscript, supervised the project.; d Consultant, Resource Center for Rare Oral Diseases, Copenhagen University Hospital, Denmark. Concept and methodology, collected data, reviewed and edited the manuscript, project administration and supervision.

**Keywords:** dental implants, implant success, implant survival, osteogenesis imperfecta

## Abstract

**Purpose:**

To investigate the survival rate of dental implants in patients diagnosed with osteogenesis imperfecta (OI).

**Materials and Methods:**

The study is a retrospective analysis of six individuals (2 males, 4 females) with OI (type I, III and IV) with a total of 25 dental implants. Clinical examination included plaque index, gingival index, periodontal pocket depth for each implant, presence of pus, and loosening of the implant(s). Marginal bone loss was measured on radiographs. The observation period ranged from 2–17 years (mean:7.5 years, median: 5 years).

**Results:**

The overall implant survival rate was 80%. One patient with OI type III lost five implants. However, four out of five lost implants functioned for 11 years.

**Conclusion:**

Dental implant treatment seems to be a valid option for replacing missing teeth in OI patients. It is recommended that patients diagnosed with OI undergo the same preoperative evaluation as regular dental implant patients with special emphasis on a healthy periodontal status and ideal oral hygiene.

Osteogenesis imperfecta (OI) is a rare hereditary connective tissue disorder with an estimated prevalence of 10.6/100,000.^1^ OI follows a dominant or recessive inheritance pattern. Autosomal dominant mutations in the genes COLIA1 and COLIA2, localised on chromosomes 7 and 17, are reported to account for 90% of cases of OI.^20^ In the remaining 10%, the disease results from mutations in other genes.^20^ Clinically, OI is characterised by enhanced bone fragility, growth impairment and bone deformities. These clinical manifestations are caused by a deficiency in the synthetisation of type I collagen, which is the most abundant protein in bone matrix and provides the bone matrix with both flexibility and strength.^7,8^ The majority of patients with OI demonstrate either quantitative or qualitative deficiences in collagen type I structure and mineralisation.

In 1979, Silence et al^28^ classified OI into four categories encompassing almost 90% of OI cases. Since then, additional subtypes have been added in parallel with identification of new mutations. The characteristics of types I-IV are described in Table 1.

**Table 1:** Charecteristics of osteogenesis imperfecta type I – IV^5^


Type I	Reduced amount of normally structured collagen type I. None to a mild degree of bone deformities and only few fractures of the long bones. Blue sclera.
Type II	Type II is lethal and is characterised by a qualitatively defective collagen type I. Death occurs prenatally or early in life due to bone fractures and respiratory failure.
Type III	Type III comprises a large variation of clinical manifestations caused by qualitatively compromised collagen Ieading to multiple fractures, bone deformities and growth impairment.
Type IV	OI type IV is an intermediate form, ranging between type I and III but does not fulfil all the characteristics related to these OI subtypes. Body height is often below average and the fracture rate varies. This type of OI may be associated with either qualitatively or quantitatively defective collagen type I.

Currently, no causative treatment is available for OI. Hence, treatment of patients with OI focuses on handling symptoms such as fractures, deformities and pain. Patients with severe forms of OI often receive prophylactic treatment with antiresorptive medication, e.g. bisphosphonates. The resulting increased bone density reduces pain, but evidence that this treatment can reduce the number of fractures and deformities is limited.^11^

In the oral cavity, malocclusion, tooth agenesis and dentinogenesis imperfecta (DI) are frequently observed in patients diagnosed with OI.^18^ DI is a condition affecting the normal development of dentin. DI is classified into types I, II and III. DI type I is associated with OI and is caused by abnormal collagen formation.^2^ DI can be seen in all types of OI, but it is most often related to types III and IV, and is rarely seen in OI type I.^27^ DI is characterised by varying degrees of yellow/brown/grey discolouration of the crown, early obliteration of the pulp canal, cervical constriction, fracture of enamel and pronounced attrition. Furthermore, cervical fracture of teeth is a frequent complication resulting in early tooth loss.^22^ Hence, edentulism causing reduced masticatory function and displeasing aesthetic dental appearance may potentially lead to a decreased quality of life.^10^

Over the past 40 years, implant therapy has evolved into a predictable standard of care to replace missing teeth and thereby improve masticatory function and facial aesthetics. In this context, implant survival and success are important. The four most frequently used parameters for assessing the success of dental implant treatment are related to peri-implant bone level, healthy peri-implant mucosa, technical complications related to implant-supported dental prosthesis and patient-reported outcome measures (PROMs).^23^

Patients with OI could potentially benefit profoundly from predictable implant treatment to replace missing teeth. However, alteration in bone quality and quantity (and antiresorptive medication) may potentially affect implant survival. OI is a rare disease, and as a consequence, implant survival and successful implant therapy in patients with OI have only been reported in a single case series and few case reports.^3,5,9,14,15,21,24,25,31,32^

Therefore, the aim of the present study was to report on six patients diagnosed with OI types I, III and IV who received oral rehabilitation, with dental implants with a focus on implant survival and success.

## Materials and Methods

This is a retrospective analysis of implant survival in patients with OI. During the period of 2001 to 2018, 82 individuals diagnosed with OI were referred to the Resource Centre for Rare Oral Diseases, Copenhagen University Hospital, Denmark. Of these, six patients received treatment with dental implants. The observation period ranged from 2–17 years (mean = 7.5 years, median: 5 years). The patients all gave informed consent to participate in the study.

The study was performed in accordance with the declaration of Helsinki and current Research Committee Regulations, and was approved by the Danish Data Protection Agency (VD-2018-318 – 6566) and the local authorities from the Capital Region of Denmark. By Danish law, this study is considered a “quality assurance follow-up study” (all data were obtained in a clinical context and/or as part of a standardised treatment protocol), and thus does not qualify for evaluation by a research ethics committee in Denmark.

From each patient’s medical record, the following data were extracted: gender, age, medication (including antiresorptive medication), smoking habits (daily smoker/non-smoker), type of OI (I-IV), tooth agenesis, number and location of dental implants.

Periodontal disease was evaluated by measuring marginal bone loss on all teeth present at the time of implant placement. Measurements were performed with a high-resolution computer monitor in a darkened room. Each tooth was measured at the site with the most pronounced bone loss. Measurements were made from the marginal bone crest to the tooth apex (total bone height) and from the cementoenamel junction to the tooth apex (total root length). The arithmetic mean, calculated from the total root length and bone height, was used as a measure of the proportion of remaining bone height supporting each tooth. Measurements were made of all teeth with visible cementoenamel junctions and visible apices. Dental implants were not examined. Based on the mean value of all teeth, participants were subsequently allocated to the following groups: healthy (≥80% remaining bone), mild to moderate periodontitis (from 79% to 66%), and severe periodontitis (<66%).^26^

The diagnosis DI was based on the presence of the following clinical and radiological signs: increased translucency of enamel, greyish-blue to brown discolouration of teeth, early and advanced or total pulp obliteration of fully developed teeth, short roots, and cervical constriction.^30^

All surgical procedures were performed according to standard protocol, and prosthetic procedures were performed according to the manufacturer’s recommendations. All patients were preoperatively premedicated with antibiotics and analgesics. Implant surgical procedures were performed under local anaesthesia. All implants were placed with or without simultaneous bone augmentation after elevation of a full-thickness mucoperiosteal flap followed by transmucosal healing. Patients were instructed to rinse with 0.12% chlorhexidine solution (twice daily until suture removal) and were administered antibiotics and analgesics. Sutures were removed 7-10 days later. Implants healed 4–5 months prior to impression and prosthetic reconstruction.

At the final follow-up, clinical examination included recording the plaque index (PI), gingival index (GI) and probing pocket depth (PPD) at four locations around each implant, including any presence of pus and/or mobility of the dental implant(s).^17^ Radiographic examination included a panoramic radiograph and periapical radiographs of implant sites. The known implant length was used for calibration of vertical bone measurements on each radiograph.^6^ Bone loss was measured mesially and distally as the distance from the implant shoulder to the first bone-to-implant contact, and the highest value was recorded for each implant. The program Bluebeam Revu (Bluebeam Revu Mac Version 1.9.3, 2018; Pasadena, CA, USA) was used for calibration and measurements. Patients’ experience of implant treatment was evaluated using the Oral Health Impact Profile (OHIP-49) questionnaire.^15^ The OHIP is a 49-item, self-administered questionnaire used for assessing the impact of oral health, masticatory ability and psychosocial function on quality of life. For the present study, special emphasis was given to questions addressing facial aesthetic appearance and masticatory function in relation to patients’ dental implants. Finally, patients were asked about their smoking habits.

## Results

The patient’s demographics are summarized in Table 2.

**Table 2:** Patient characteristics

Patient	1	2	3	4	5	6
Gender	M	F	F	F	F	M
Age at final examination	49	42	43	57	25	64
OI type	I	III	III	IV	IV	IV
Antiresorptive medication	+	+	–	+	+	–
Dentinogenesis imperfecta	–	–	+	+	+	–
Dental agenesis*	–	–	–	–	⁺	–
Periodontal disease**	Severe	Mild to moderate	Severe	Mild to moderate	Healthy	Healthy
+/– Bone augmentation	+	–	+	+	+	–
+/– smoking***	+	–	–	–	–	–
Number of implants placed	4	3	5	8	1	4
Implants lost	0	0	5	0	0	0

*Agenesis in the dentition; **healthy ≥ 80% remaining bone, mild to moderate 21–33% remaining bone, severe < 66% remaining bone; ***smoking on a daily basis.

A total of 25 implants were placed in six patients. At the final examination, 20 implants in five patients were still functioning, whereas one patient (patient #3) had lost all five of her implants, yielding an overall implant survival rate of 80%. It should be noted that this patient had an early, pre-loading implant failure of one of her four inserted implants (at location 44), and therefore a new, larger-diameter implant was inserted at the same location, for a total of five dental implants. The patient also lost this new implant, which explains the total loss of five implants.

The findings of the radiographic examination are summarized in Table 3. All implants demonstrated a bone loss of 3 mm or less after 2 to 17 years of functional loading. Details of the prosthetic reconstructions are given in Table 4.

**Table 3:** Findings of the radiographic examinations

Patient	OI Type	Location of implant	Brand	Implant length (mm)	Bone loss (mm)	Implants lost	Years of follow-up
1	II						
		17	AS	13	2	No	4
		16	“	13	0	No	5
		15	“	13	1	No	5
		26	“	13	0	No	4
2	III						
		42	AS	11	0	No	2
		41	“	11	0	No	2
		32	“	11	0	No	2
3	III						
		44	N	11,5	n/a	Yes	0
		44	“	11,5	n/a	Yes	11
		42	“	11,5	n/a	Yes	11
		32	“	11,5	n/a	Yes	11
		34	“	11,5	n/a	Yes	11
4	IV						
		11	N	13	0	No	5
		21	AT	12	0	No	17
		22	N	16	0	No	13
		25	AT	11	0	No	5
		45	AT	9	1	No	10
		44	AT	13	1	No	10
		34	AT	12	1	No	12
		35	AT	9	3	No	12
5	IV						
		35	ST	10	1	No	4
6	IV						
		15	SB	10	1	No	4
		14	SB	12	0	No	4
		24	AS	12	0	No	4
		25	AS	10	0	No	4

AS: Astra Osseospeed; AT: Astra Tioblast; N: Nobel Biocare; ST: Straumann Tissue Level SLA; SB: Straumann Bone Level SLA.

**Table 4:** Prosthetic restorations in patients with osteogenesis imperfecta

Patient	Number of implants	Prosthetic restoration
1	4	Four implant-supported single crowns, of which 3 crowns in the right maxilla were soldered together
2	3	4-unit fixed dental prosthesis
3	4	Full-arch fixed dental prosthesis
4	8	Eight implant-supported single crowns, of which two implants in each side of the mandible were soldered together
5	1	Implant-supported single crown
6	4	Four implant-supported single crowns

The clinical examination revealed healthy peri-implant conditions with 93% of all implant surfaces free of plaque and 94% free of mucositis. In all cases, PPD was below 6 mm, and no pus was observed. All implants were stable. No signs of bruxism were recorded. Concerning smoking, one patient smoked five cigarettes per day. Two patients were previous smokers but quit before dental implant placement.

## Discussion

Clinically and radiographically successful implant treatment could be documented in five out of six patients after up to 17 years of function. This result compares well with previous publications reporting on cases of patients with OI receiving dental implants (Table 5). In these eight case reports and one retrospective and prospective case series, a total of 107 implants were placed in 21 patients with 1-12 years of follow-up.^5,6,9,14,15,21,24,25,31,32^ As in the present study, the majority of patients (n=6) obtained well-integrated implants with healthy conditions.

**Table 5:** Scientific articles on patients with OI rehabilitated with dental implants

Author, reference number	Year of publication	Gender	OI type	Number of implants	+/- Bone augmentation	Number of implants lost	Years of follow-up
Zola^32^	2000	M	n/a	19	+	7	7
Binger et al^3^	2006	F	n/a	5	+	0	5
Lee and Ertel^16^	2003	F	III	2	+	0	2
Prabhu et al^25^	2007	M	IV	11	-	1	2
Payne et al^24^	2008	F	IV	11	+	0	2
Wannfors et al^31^	2009	F	III	4	+	0	3
Friberg^9^	2013	F	n/a	6	-	0	4
Caicedo-Rubio et al^5^	2017	M	IV	3	-	0	4
Jensen et al^15^/Myint et al^21^	2011/2019						
1		F	I	1	-	0	10
2		M	I	5	+	0	12
3		M	I	5	-	0	5
4		M	IV	1	-	0	7
5		F	I	6	-	0	8
6		F	III	7	-	2	5
7		M	I	7	-	0	5
8		F	I	2	-	0	2
9		M	I	5	-	0	9
10		M	III	1	-	0	2
11		M	I	3	-	0	8
12		F	I	2	-	0	7
13		F	IV	1	-	0	7
Fogh et al	2023 (this article)						
1		M	I	4	+	0	5
2		F	III	3	-	0	2
3		F	III	5	+	5	11
4		F	IV	8	+	0	17
5		F	IV	1	+	0	4
6		M	IV	4	-	0	4

Including the present study, existent literature hence describes the result of a total of 132 implants placed in 27 patients with OI. Four patients (15%) lost 15 implants (11%) after 1 month to 17 years of loading. A survival rate of 89% is slightly below the rates reported for implants placed in healthy individuals.^4^

Several factors may affect the prognosis and lead to implant failure in patients with OI. In patients with OI, especially OI type III, the bone is characterised by an abundance of woven bone and less mature lamellar bone.^18^ It may be speculated that this type of bone provides less than ideal conditions for long-term implant function. However, no histological studies have yet reported on bone quality at specific dental implant sites. The patient from the present study who lost all five implants suffered from OI type III. One month after placement of four implants, the first implant was mobile and was removed. Two months later, a new implant was inserted at the same place. After 11 years of follow-up, all four implants were lost. The implants were placed in the anterior mandible where the alveolar ridge is narrow and extremely compact. Early loss of implants (before placement of abutment) was also reported in a previous study, in a patient with type III OI.^14^ However, one other patient from the present case series and three previously reported cases^14,15,31^ was diagnosed with OI type III without experiencing implant failure after 5 years of follow-up.

OI patients are often treated with antiresorptive medication in the form of low-dose bisphosphonates to reduce the risk of bone fractures. Low-dose bisphosphonates are not considered a contraindication for dental implant therapy.^29^ However, long-term administration of low-dose bisphosphonates may increase the risk of medication-related osteonecrosis of the jaws (MRONJ).^29^ OI patients may be treated with bisphosphonates from early childhood and have therefore most often already received their medication long-term when implants are placed. Interestingly, the only patients that lost implants in the present case series had not been treated with bisphoshonates.

Patients with OI often experience difficulties performing sufficient oral hygiene. Ideal oral hygiene is in general considered a prerequisite to be considered a candidate for dental implants.^16^ Implant failure due to compromised oral hygiene in patients with OI should thus be considered a side-effect rather than a direct result of the OI condition. It should be emphasised that all patients who retained their dental implants in the present study had excellent oral hygiene and healthy periodontal conditions. On the other hand, the patient who lost all of her five implants also lost her teeth due to periodontal disease. It is well established that active periodontal disease predisposes to implant loss.^16^ Interestingly, the patient reported by Zola^32^ who lost 7 of 19 implants was also diagnosed with periodontal disease.

None of the patients in the present study were enrolled in a systematic periodontal maintenance programme. Similarly, no maintenance programme was described in the previously reported cases.

Based on these findings, it is recommended that implant treatment planning in patients with OI follow the same guidelines as in patients without OI. Hence, if the patient presents with active periodontal disease, the patient should undergo supportive periodontal therapy before placement of dental implants is considered.

However, even with a potentially lower long-term survival rate, patients diagnosed with OI can possibly benefit significantly from dental implants for several years, as seen in patient #3 in the present study, and dental implants should therefore be considered as a treatment option.

Limitations of the present study include the limited number of patients, the heterogeneous nature of the patient group, the retrospective nature of the study and the relatively short follow-up period for some of the included patients. OI is a rare disease; we therefore recommend that future research be conducted as prospective, multicentre studies to include a larger number of patients. Furthermore, future studies should focus on histological bone quality in the different types of OI and aim to relate this information to implant survival.

## Conclusions

Dental implant treatment seems to be a valid option to replace missing teeth and restore masticatory function and aesthetics in patients with OI. It is recommended that patients diagnosed with OI undergo the same preoperative evaluation as other dental patients with respect to general health status, medication, smoking habits, periodontal disease and oral hygiene. The entire handling of oral health for patients diagnosed with OI is complex due to the disease characteristics, potential malocclusion and DI. It is therefore recommended that treatment planning and treatment be provided by specialised units.
